# The novel function of an orphan pheromone receptor reveals the sensory specializations of two potential distinct types of sex pheromones in noctuid moth

**DOI:** 10.1007/s00018-024-05303-2

**Published:** 2024-06-15

**Authors:** Chenrui Wang, Song Cao, Chen Shi, Mengbo Guo, Dongdong Sun, Zheyi Liu, Peng Xiu, Yong Wang, Guirong Wang, Yang Liu

**Affiliations:** 1 State Key Laboratory for Biology of Plant Diseases and Insect Pests, Institute of Plant Protection, Chinese Academy of Agricultural Sciences, Beijing, 100193 China; 2 Shenzhen Branch, Guangdong Laboratory for Lingnan Modern Agriculture, Genome Analysis Laboratory of the Ministry of Agriculture and Rural Affairs, Agricultural Genomics Institute at Shenzhen, Chinese Academy of Agricultural Sciences, Shenzhen, 518120 China; 3 Institute of Evolution and Ecology, School of Life Sciences, Central China Normal University, Wuhan, 430079 China; 4 Department of Engineering Mechanics, Zhejiang University, Hangzhou, 310027 China; 5 Department of Plant Protection, Advanced College of Agricultural Sciences, Zhejiang A & F University, Hangzhou, 311300 China; 6 College of Life Sciences, Zhejiang University, Hangzhou, 310058 China; 7 The Provincial International Science and Technology Cooperation Base on Engineering Biology, International Campus of Zhejiang University, Haining, 314499 China

**Keywords:** *Helicoverpa armigera*, Type II sex pheromone, Pheromone receptor, Olfactory sensilla, Molecular docking

## Abstract

Sex pheromones play crucial role in mating behavior of moths, involving intricate recognition mechanisms. While insect chemical biology has extensively studied type I pheromones, type II pheromones remain largely unexplored. This study focused on *Helicoverpa armigera*, a representative species of noctuid moth, aiming to reassess its sex pheromone composition. Our research unveiled two previously unidentified candidate type II sex pheromones—3Z,6Z,9Z-21:H and 3Z,6Z,9Z-23:H—in *H. armigera*. Furthermore, we identified HarmOR11 as an orphan pheromone receptor of 3Z,6Z,9Z-21:H. Through AlphaFold2 structural prediction, molecular docking, and molecular dynamics simulations, we elucidated the structural basis and key residues governing the sensory nuances of both type I and type II pheromone receptors, particularly HarmOR11 and HarmOR13. This study not only reveals the presence and recognition of candidate type II pheromones in a noctuid moth, but also establishes a comprehensive structural framework for PRs, contributing to the understanding of connections between evolutionary adaptations and the emergence of new pheromone types.

**Supplementary Information:**

The online version contains supplementary material available at 10.1007/s00018-024-05303-2.

## Introduction

Mate attraction in numerous insects is facilitated through pheromones, with the most extensively studied instance being the utilization of sex pheromones in Lepidopteran moths. Typically, females release species-specific pulses of a sex-pheromone blend that are both emitted and alluring to conspecific males across considerable distances [1]. Since the identification of the first insect sex pheromone, bombykol, (*E*, *Z*)-10,12-hexadecadien-1-ol (E10,Z12-16:OH; subsequent abbreviations follow the same pattern), found in females of the silkworm moth *Bombyx mori* [2, 3], sex pheromones have been elucidated in approximately 700 moth species [4, 5]. However, it is crucial to recognize that our understanding in this field remains restricted, given the extensive species diversity within the Lepidopteran moth family.

Lepidopteran sex pheromones are categorized into two primary groups (Types I and II) and two additional miscellaneous groups (Types 0 and III) based on their distinct chemical structures [4, 6]. Type I pheromones consist of unsaturated C10–C18 fatty alcohols and their derivatives (primarily acetates and aldehydes) with long, straight chains, constituting approximately 75% of all reported moth pheromones. Type II pheromones comprise C17–C25 unbranched polyunsaturated hydrocarbons and their corresponding epoxide derivatives, characterized by longer straight chains, and are employed by about 15% of reported moth species [6]. Despite being synthesized through different pathways and characterized by variations in biosynthetic sites, substrate materials, biosynthetic enzyme systems, and endocrine regulation mechanisms [1, 4, 6], both primary types of sex pheromones serve a congruent function in facilitating mate recognition and attraction in moths.

To date, most of research efforts have been focused on type I pheromones, with type II pheromones receiving relatively less attention, alone the other two types. Type II pheromones in Lepidoptera were first identified in females of the artiine moth *Utetheisa ornatrix*, and they appear to be restricted to a few moth families, including Tischeriidae, Crambidae, Geometridae, and Erebidae [6]. In general, each moth species utilizes only one type of pheromone; for instance, the silkworm moth uses type I pheromones [2, 7], while the winter moth *Operophtera brumata* employs type II pheromones [8]. However, recent studies suggest that an increasing number of moth species employ blends of type I and type II compounds as sex pheromones, a hybrid pheromone system. This phenomenon has been observed in the families of Crambidae [9–12], Pyralidae [13–15], Tortricidae [16], Erebidae [17], and Noctuidae [18], indicating that such combinations may be more widespread than previously thought. Furthermore, in most cases, type I pheromones serve as the major component for long-range attraction, while type II pheromones, despite being the minor component, play a significant role in close-range contact and the facilitation of mating behaviors [10, 16, 19, 20].

In the male response to conspecific sex pheromones, the selectivity and specificity of sex pheromone reception are determined by pheromone receptors (PRs) expressed on the dendrites of specific olfactory receptor neurons (ORNs) in antennal sensilla. These PRs, as specialized members of the insect odorant receptor (OR) family, possess a seven-transmembrane structure and form heteromeric ligand-gated non-selective cation channels in association with the odorant receptor co-receptor (Orco) [21, 22]. Given to their direct interactions with pheromone molecules and their role in initiating the signal transduction process, PRs have become pivotal targets in the study of moth pheromone recognition systems. Numerous studies have been conducted to identify and functionally de-orphanize PRs in moths [23–25]. However, it is essential to note that the majority of characterized PRs respond to type I pheromones [23, 24], with only two PRs identified as responsive to type II pheromones [26, 27]. The first of these PRs, ObruOR1 from the winter moth *O. brumata*, specifically responds to its major pheromone component, 1,3Z,6Z,9Z-21:H [8, 27]. Additionally, its orthologue, AsegOR3 in *Agrotis segetum*, is strongly activated by another type II hydrocarbon, 3Z,6Z,9Z-21:H [27] The second PR, EgriOR31, identified in the tea geometrid, *Ectropis grisescens*, is highly selective for 3Z,9Z-6,7-epo-18:H [26], the major pheromone component of *E. grisescens* [28]. Despite both PRs binding to type II pheromones, their ligands exhibit notable differences, particularly in carbon chain lengths and functional groups. Furthermore, molecular phylogenetic analyses reveal that EgriOR31 is separated from the traditional PR subfamily [26], where ObruOR1 and AsegOR3 cluster, indicating that they have evolved from two independent ancestral genes.

The cotton bollworm, *Helicoverpa armigera*, is one of the most destructive agricultural pests worldwide, infesting a wide range of crops [29]. The sex pheromones of the species are a blend of several type I pheromone components, featuring a predominant compound, Z11-16:Ald, along with several minor compounds like Z9-16:Ald, Z9-14:Ald, etc [30, 31]. Studies have shown that a mixture of Z11-16:Ald and Z9-16:Ald with a ratio from 99:1 to 90:10, significantly enhances male *H. armigera* trap catches [30, 32]. Z9-14:Ald, on the other hand, exhibits contrasting behavioral effects at different concentrations when combined with other pheromone components [33]. Moreover, Z11-16:OH functions as a pheromone antagonist, regulating the optimal mating time for *H. armigera* [34].

Previous investigations have identified seven PR genes, namely *HarmOR6*, *HarmOR11*, *HarmOR13*, *HarmOR14*, *HarmOR14b*, *HarmOR15*, and *HarmOR16*, by analyzing antennal transcriptome data [35, 36]. These genes have undergone extensive functional characterization in subsequent investigations using both in vivo and in vitro methods [25, 34, 37–42]. Despite ongoing debates regarding the final conclusions, all these PRs have demonstrated activation in response to type I pheromones, except for HarmOR11. This particular receptor, highly expressed in both male and female antennae [35, 37], dose not display affinity for the type I pheromones tested.

In this study, we identified two candidate type II pheromone components, 3Z,6Z,9Z-21:H and 3Z,6Z,9Z-23:H in adult *H. armigera*. Through a combination of phylogenetic analysis, genome editing, and electrophysiology techniques, we established that an orphan PR, HarmOR11, functions as a receptor for 3Z,6Z,9Z-21:H, which is notably abundant in the male abdomen. Subsequently, we elucidated the mechanisms governing the diversification of sensory specializations in HarmOR11 and HarmOR13, a receptor of type I pheromone using molecular dynamics (MD) simulations for each PR based on AlphaFold2 structural prediction and molecular docking. Our study not only unveils the presence and perception of candidate type II pheromones in *H. armigera*, but also establishes a foundation for understanding the structural basis of PRs responsible for different pheromone types.

## Materials and methods

### Insects

*H. armigera* used in all experiments were reared at the Institute of Plant Protection, Chinese Academy of Agricultural Sciences, Beijing, China. The larvae from all strains were fed on an artificial diet under conditions of 26 ± 1 ℃, 60 ± 5% relative humidity (RH), and a photoperiod of 14:10 h (L: D). Pupae were sexed and kept in individual glass tubes for eclosion. Adult moths were provided a daily diet of 10% sucrose solution for sustenance.

### Chemical compounds

(−)-β-Pinene, cis-Jasmone, benzaldehyde, salicylaldehyde, geraniol and (Z)-2-Hexen-1-ol were purchased from Sigma-Aldrich (Saint Louis, Missouri, USA). (Z)-11-hexadecenal (Z11-16:Ald), (Z)-9-hexadecenal (Z9-16:Ald), (Z)-9-tetradecenal (Z9-14:Ald) and (Z)-11-hexadecen-1-ol (Z11-16:OH) all with the purity above 95%, were purchased from Nimrod Inc. (Changzhou, China). The polyenes, 3Z,6Z,9Z-nonadecatriene (3Z,6Z,9Z-19:H), 3Z,6Z,9Z-heneicosatriene (3Z,6Z,9Z-21:H), 3Z,6Z,9Z-tricosatriene (Z3,Z6,Z9-23:H) and 3Z,6Z,9Z,12Z,15Z-pentacosapentaene (3Z,6Z,9Z,12Z,15Z-25:H) were purchased from Pherobank BV, Wijk bij Duurstede, The Netherlands (listed in Supplementary Table S1). For the two-electrode voltage-clamp (TEVC) recordings assay, the polyenes were dissolved in dimethyl sulfoxide (DMSO) to prepare 1 mol stock solution and stored at -20 °C until use. Prior to usage, the stock solution was diluted into 1×Ringer’s buffer (96 mmol NaCl, 5mmol MgCl_2_, 2 mmol KCl, and 5mmol HEPES, pH = 7.6) supplemented with 600 µmol of CaCl_2_ to desired concentrations. For other electrophysiological experiments, the chemicals were dissolved in hexane to concentrations of 10 µg µL^− 1^ and 100 µg µL^− 1^, and stored at -20 °C until required.

### Chemical extracts

The pheromone glands of 2- to 3-day-old virgin females and males were dissected 4–6 h into the scotophase and soaked in hexane solvent for 30 min in ambient temperature. Following the removal of the pheromone glands, the abdomen was detached from the thorax at the junction and cut laterally [43]. The excess fat and internal organs were discarded carefully. The abdominal cuticle along with the epidermal tissue then was immersed in hexane at room temperature for 30 min. To obtain enough amount of potential chemical compounds, 10 and 6 pheromone glands from females and males, respectively, were pooled in a single glass vial (2 mL, Agilent Technologies, Santa Clara, CA, USA) containing 300 µL of hexane. In contrast, both 5 abdomen tissues of each sex were soaked in 1 mL of hexane, respectively. After a straightforward filtration process, the crude pheromone gland extracts and abdomen extracts were transferred into a new glass vial with a 250 µL glass insert (Agilent, Santa Clara, CA, USA), and all samples were concentrated to 20 µL under a gentle stream of nitrogen. Concentrated extracts were kept in -20 ℃ before used in chemical analysis.

### Gas chromatography coupled with mass spectrometry (GC-MS) analysis

Pheromone gland extracts or abdomen extracts were subjected to GC-MS analysis using a QP2020 SE gas chromatograph (GC) coupled with a capillary column Rtx-5MS (30 m, 0.25 mm inner diameter, 0.25 μm film thickness, Shimadzu, Tokyo, Japan). The non-shunt injection method was employed, utilizing helium gas as the carrier, with a flow rate of 3 mL min^− 1^. The inlet temperature was 250 ℃ with the initial temperature of the column set at 40 ℃. The GC was programmed from starting at 40 ℃ for 1 min, then ramping up to 200 ℃ at 15 ℃ min^− 1^, then to 215 ℃ at 3 ℃ min^− 1^, and finally to 260 ℃ at 5 ℃ min^− 1^ with a final hold for 5 min. The interface temperature was maintained at 260 ℃, while the ion source of the detector was set at 250 ℃. A solvent delay time of 3 min was implemented, and the mass scan range spanned from 35 to 500 *m/z*. The mass spectra were taken in electron ionization (EI) mode at 70 eV. A manual injection of 1 µL was administered each time. This identical procedure was executed for both the test samples and the standard samples. To determine the content of candidate polyenes in each tissue, we used GC-MS to establish standard curves of the synthetic chemicals with gradient dosages under the same conditions.

### RNA isolation and cDNA synthesis

RNA was extracted from the antennae of *H. armigera* utilizing Trizol regent (Invitrogen, USA). The homogenized mixture was enveloped with 1 mL of Trizol reagent, and the subsequent extraction of total RNA was performed according to the manufacturer’s instructions. RNA was dissolved in RNase-free H_2_O, and its integrity was verified via gel electrophoresis. The amount of RNA was determined on a Nanodrop ND-1000 spectrophotometer (NanoDrop products, USA). Subsequently, cDNA synthesis was undertaken employing the Revert Aid First Strand cDNA Amplification Kit (Thermo Fisher, USA), and the synthesized cDNA was stored at -20 ℃ and further used for gene cloning.

### Gene cloning

The full-length sequences of *HarmOR11* (accession number MN399771) and *HarmOrco* (accession number MN399769) were amplified with the gene-specific primers of each gene. PrimeSTAR HS (Premix) (Takara, Japan) was used to amplify the two genes within a 25 µL PCR reaction volume respectively, containing 12.5 µL of 2×PrimeSTAR HS Premix, 1 µL of each primer, and 1 µL of cDNA template. The PCR was performed under the conditions of 98 ℃ for 3 min, 35 cycles of 98 ℃ for 10 s, 57 ℃ for 5 s, and 72 ℃ 40 s, followed by a final extension of 72 ℃ for 10 min. The PCR products were separated by electrophoresis on 1.0% agarose gels and the target DNA bands were purified using the EASYPure Quick Gel Extraction Kit (TransGen, Beijing, China). The purified products were subsequently ligated into the pEASY-Blunt Cloning Vector (TransGen, Beijing, China) and transformed into *Trans5α* Chemically Competent Cell (TransGen, Beijing, China). Positive colonies were confirmed via Sanger sequencing (Sangon Biotech, Shanghai, China). The verified sequences of the two genes were further subcloned into an expression vector pT7Ts with primers bearing a restriction site and a Kozak sequence to the 5’ end, as well as a distinct restriction site to the 3’ end. The primers employed for gene cloning and vector construction were listed in Supplementary Table S2.

### Phylogenetic analysis

Alignments of amino acid sequences were performed by MAFFT version 7. A phylogenetic tree was constructed using RAxML version 8 with the Jones–Taylor–Thornton (JTT) amino acid substitution model [44]. Node support was assessed using a bootstrap method based on 1000 replicates. The phylogenetic tree was constructed using a total of 183 OR sequences from five Lepidoptera species: 57 from *H. armigera*, 60 from *Spodoptera littoralis* [45], 62 from *B. mori* [46], 2 from *A. segetum* and 2 from *O. brumata* [27]. Dendrograms were created and colour labeled with FigTree v1.4 software (http://tree.bio.ed.ac.uk/software/figtree/).

### Screening of HarmOR11 mutant strain

The nucleotide sequence of *HarmOR11* was subjected to a BLAST search against the genome sequence of *H. armigera* under the accession number GCA_030705265.1 [47] to determine the exon-intron regions within *HarmOR11*. The loci information of *HarmOR11* was further visualized using the online tool GSDS 2.0 (http://gsds.gao-lab.org/).

According to the principle of 5’-N_19_NGG-3’, a conserved nucleotide sequence, 5’-TCTATCTCAGAAATATAGAGAGG**-**3’, situated in exon 3 was chosen as sgRNA target site. Extensive analysis revealed no potential off-target effects in other genomic regions. The synthesis of the sgRNA was carried out using GeneArt Precision gRNA Synthesis Kit (ThermoFisher Scientific, USA), following the manufacturer’s instructions.

Fresh eggs were collected at hourly intervals and affixed to microscope slides using double-sided adhesive tape. Each egg was individually injected with a mixture of sgRNA (300 ng µL^− 1^) and Cas9 protein (200 ng µL^− 1^; Invitrogen TrueCut Cas9 Protein v2) via the FemtoJet and injectMan NI 2 Microinjection System (Eppendorf, Hamburg, Germany). These injected eggs were maintained in sterile Petri dishes, adhering to normal rearing conditions conducive to hatching. Once hatched, the larvae were fed with artificial diet before pupation. After eclosion, a middle leg from each individual was carefully dissected to facilitate gDNA extraction using TIANamp Genomic DNA Kit (TIANGEN, Beijing, China). Subsequently, a target DNA fragment encompassing the target site was amplified with a pair of gene specific primers (listed in Supplementary Table S2). PCR was performed in 25 µL volumes containing 12.5 µL of 2×PrimeSTAR HS Premix, 1 µL of each primer, 2 µL of gDNA template, and 8.5 µL of ddH_2_O, under the following conditions: 98 ℃ for 3 min, 35 cycles of 98 ℃ for 10 s, 57 ℃ for 5 s, and 72 ℃ 30 s, with a final extension of 72 ℃ for 5 min. PCR products were further sequenced with the forward primer, and multiple peaks were decoded by the Degenerate Sequence Decoding (DSD) method to determine genotypes [48]. The moths carrying the same mutation were mixed to obtain homozygous mutant in G1.

### In situ hybridization

Digoxigenin (DIG)-labelled sense and antisense RNA probes of *HarmOR11* were synthesized using the T7/SP6 RNA transcription system, following the manufacturer’s protocol (Roche, Basel, Switzerland). RNA probes were fragmented to an average length of around 200 bp by incubation in fragmentation buffer (120 mM Na_2_CO_3_, 80 mM NaHCO_3_, pH 10.2) at 60 °C.

Antennae from male and female moths aged 2 to 3 days were embedded in Jung tissue-freezing medium (Leica, Nussloch, Germany) and frozen at -22 °C on an object holder. Cryosections (12 μm) were sectioned with a cryostat microtome (Leica CM1850, Germany), thaw-mounted on anti-off slides and air-dried at room temperature for 30 min. In situ hybridization was performed followed established procedures as outlined in a prior study [49]. For the visualization of cells expressing *HarmOR11*, a DIG-labelled antisense probe specific to *HarmOR11* was applied to the antenna sections of male and female moths. As a control, a sense probe was also utilized. Cryosections were examined on an Olympus microscope (Olympus, Tokyo, Japan) equipped with Cellsens Dimension software.

### HarmOR11 expression in Xenopus oocytes and electrophysiological recordings

The TEVC recording technique combined with the *Xenopus* oocyte heterologous expression system has been widely employed to deorphanize the ligands of insect ORs [50]. Capped RNA (cRNA) for each gene was synthesized from a linearized vector using the mMESSAGE mMACHINE T7 Kit (Ambion, Austin, TX, USA), according to the manufacturer’s guidelines. Oocytes were surgically obtained from mature female *Xenopus laevis* frogs. After treated with 2 mg mL^− 1^ of collagenase I in 1×Ringer’s buffer supplemented with 10 µg mL^− 1^ of gentamycin, oocytes of good quality were singled out and microinjected with a mixture comprising 27.6 ng of HarmOR11 cRNA and 27.6 ng of HarmOrco cRNA. Post-injection, the oocytes were incubated at 18 °C in 1×Ringer’s buffer supplemented with 5% dialysed horse serum, 550 µg mL^− 1^ of sodium pyruvate, 100 µg mL^− 1^ of streptomycin, 50 µg mL^− 1^ of tetracycline, and 600 µmol of CaCl_2_. After 3–5 days of incubation, the responses of oocytes expressing HarmOR11 to a range of chemical compounds were recorded using an OC-725 C oocyte clamp (Warner Instruments, Hamden, CT, USA) at a holding potential of − 80 mV. The data were acquired and analyzed with Digidata 1440 A and pCLAMP 10.2 software (Axon Instruments Inc., Union City, CA, USA).

### Electroantennogram (EAG) recording

The antenna of 2–3 days old adult moths was cut at the base of the flagellum, and the tip was removed to make better contact with the glass electrode. The glass electrode was filled with 0.1 M KCl solution. To deliver the stimuli, a filter paper strip (0.3 cm × 5 cm) loaded with either 10 µL of a 10 µg µL^− 1^ or 100 µg µL^− 1^ test chemical solution, was inserted into a Pasteur pipette (15 cm long). A total of 10 µL of solvent was used as blank control. An air controller (CS-55, Syntech, Germany) was used to produce a purified and humidified continuous airflow (30 mL s^− 1^) and a stimulating flow (0.2 s at 20 mL s^− 1^). The intervals between two stimuli were above 30 s. The EAG signals were amplified using a 10×AC/DC headstage preamplifier (Syntech, Germany) and acquired with an Intelligent Data Acquisition Controller (IDAC-4- USB; Syntech, Germany). The signals were recorded and analyzed with the EAG-Pro Software (Syntech, Germany).

### Single sensillum recording (SSR)

Polyenes and other chemicals used in SSR were dissolved in hexane to working concentrations. The concentrations were set at 100 µg µL^− 1^ for polyenes and 10 µg µL^− 1^ for other chemical compounds. A filter paper strip contained 10 µL of each solution was inserted into a Pasteur pipette. Pasteur pipette carried polyene compounds were preheated to approximately 100 °C before stimulation for their less volatile.

SSR were conducted on the sensilla trichoid located on antennae of 2- to 3- day old virgin males and females. Adult moth was immobilized within a 1 mL pipette tip, allowing the head to protrude, and were fixed to the rim of the pipette tip with dental wax. One antenna was carefully removed scales and then attached to a coverslip with double-sided adhesive tape. The recording was performed under a LEICA Z16 APO microscope, with a magnification of 920×. During the recording, the reference electrode was inserted into one of the compound eyes, and the recording electrode, electrolytically sharpened into 28% KNO_2_ solution, was inserted into the base of sensilla trichoid to obtain a stable electrical signal with a high signal-to-noise ratio. Signals of the action potentials were amplified 10× using a pre-amplifier (IDAC-4 USB System; Syntech, Germany) and were displayed on a computer with the Autospike 32 software package (Syntech, Germany). Activities of each neuron were recorded for 10 s, starting 1 s prior to the delivery of a 0.3 s stimulation. The preparation was exposed to a humidified continuous airflow maintained at 20 mL s^− 1^ using a stimulus controller (CS-55, Syntech, Germany). The number of ORNs housed in a single sensillum was deduced based on spike amplitudes. The number of responses was determined by calculating the difference between the spike count 1 s before the stimulus delivery and the spike count 1 s after the stimulus was delivered.

### Molecular modelling and simulations

The prediction of heteromeric structures for HarmOrco and HarmOR11/HarmOR13, involving a stoichiometry of two ORs and two Orcos, was executed through the utilization of the AlphaFold2 (AF2) multimer [51] on our local computer cluster, implementing the default pipeline. Significantly, the AF2 prediction for the HarmOR11-Orco heterotetramer identified two distinct conformational states of the crucial gating residues F427 of OR11 and Q471 of Orco: a closed state with both residues oriented towards the center of the ion channel (State1) and another presumed open state with both residues facing outward (State2). State2 was chosen for further docking. In contrast, the AF2 predictions for the HarmOR13-Orco heterotetramer showed a consistent state with minimal conformational variation. Subsequently, the AF2 structures of HarmOR11 and HarmOR13 were employed for docking with the corresponding pheromones (3Z,6Z,9Z-21:H, PubChem ID 13116578, for HarmOR11, and Z11-16:Ald, PubChem ID 5364495, for HarmOR13, respectively) via AutoDock Vina [52]. The symmetry consideration led to identical binding modes in the two OR pockets of the heterotetrameric OR-Orco complex. The initiation of models for the pheromone-bound states was based on the selection of docked conformations with the lowest docking energy.

Subsequently, these apo and pheromone-bound models were integrated into a lipid bilayer comprising 1-palmitoyl-2-oleoyl-sn-glycero-3-phosphocholine (POPC) using PPM2.0 [53] and were solvated in a cubic water box containing 0.15 M NaCl to emulate physiological conditions. The systems were prepared using the CHARMM-GUI web server [54, 55] and underwent an energy minimization step employing the steepest descent algorithm followed by a six-step equilibration, gradually removing position constraints. The CHARMM36m force field [56] was used for the protein and POPC lipids, while the TIP3P water model for explicit solvent. Force field parameters for pheromone compounds were generated by the CHARMM General Force Field (CGenFF) [57]. In the MD simulations, temperature was maintained at a constant 310 K using a Nose–Hoover thermostat with a 1 ps coupling constant, and pressure was held at 1.0 bar with the Parrinello–Rahman barostat having a 5 ps time coupling constant. A 1.2 nm cut-off was applied for van der Waals interactions with a switch function starting at 1.0 nm. Similarly, the short-range electrostatic interactions employed a 1.2 nm cut-off, while the long-range electrostatic interactions were calculated using the particle mesh Ewald decomposition algorithm with a mesh spacing of 0.12 nm. Two 1µs semi-NPT simulations were performed for each system as production simulations, yielding data for subsequent analysis. In each simulation, there were two runs of the pheromone-bound state and one run of the apo state of the heterotetrameric OR-Orco complexes. This setup yielded a total of four replicas from two 1 µs MD simulations for the pheromone-bound state and two replicas from a 1 µs MD simulation for the apo state (Supplementary Table S3). The analysis of conformational distribution specifically concentrated on the trajectories of the last 500 ns.

All simulations were performed using a GPU-accelerated version of Gromacs 2022.5 [58]. MD trajectories were analyzed using GetContacts (https://getcontacts.github.io/), Gromacs gmx tools, and PLUMED [59]. The density maps of bound ligand were analyzed using GROmaρs [60]. The molecular structures were visualized using Pymol.

## Results

### GC/MS analyses of candidate type II sex pheromones in adult *H. armigera*

**Fig. 1:**
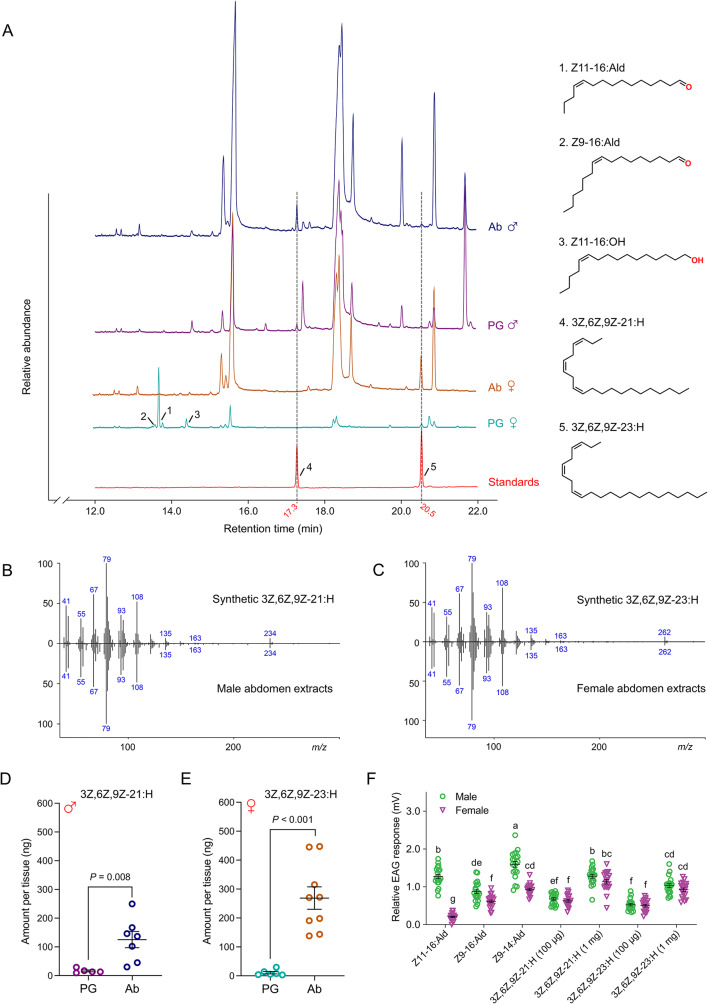
Gas chromatograph/mass spectrometry analyses of extracts in adult *Helicoverpa armigera*. **A** Left, total ion chromatogram (TIC) of female pheromone gland (PG, teal), female abdomen (Ab, rust), male pheromone gland (PG, purple), male abdomen (Ab, navy blue), and two standards (red). Right, structure and formula of five sex pheromones identified from pheromone glands and abdomens of adult *H. armigera*. **B** Mass spectra of synthetic 3Z,6Z,9Z-21:H (up) and the corresponding compound from male abdomen extracts (down). **C** Mass spectra of synthetic 3Z,6Z,9Z-23:H (up) and the corresponding compound from female abdomen extracts (down). **D** Average amount of Z3,Z6,Z9–21:H in individual pheromone gland and abdomen of male adults. Data presented are mean ± SEM (*n* = 5, 7 biological replicates), and statistical analysis was conducted by a two-tailed unpaired Student’s *t*-test. **E** Average amount of Z3,Z6,Z9–23:H in individual pheromone gland and abdomen of female adults. Data presented are mean ± SEM (*n* = 6, 9 biological replicates), and statistical analysis was conducted by a two-tailed unpaired Student’s *t*-test. **F** Relative EAG responses to sex pheromones in males (green) and females (purple). Type I pheromones were used in a concentration of 10 µg µL^− 1^, and type II pheromones were used in concentrations of 10 µg µL^− 1^ and 100 µg µL^− 1^. Data are shown as mean ± SEM (*n* = 20 biological replicates), and were analyzed by one-way ANOVA followed by two-side Tukey ’s post hoc test for multiple comparisons with SAS 9.2 for Windows. Different letters indicate significant differences (*P* < 0.05)

Using gas chromatography (GC) and GC-mass spectrometry (GC/MS), we identified sex pheromone components in the crude extracts from pheromone glands (PG) and abdomen (Ab) of adult *H. armigera*. The type I sex pheromones, including Z11-16:Ald, Z9-16:Ald, and Z11-16:OH, were identified in the female pheromone gland, as previously investigated (Fig. 1A) [34]. Additionally, two candidate type II sex pheromones, 3Z,6Z,9Z-21:H (Retention time 17.3 min) and 3Z,6Z,9Z-23:H (Retention time 20.5 min), were conclusively identified in *H. armigera* for the first time, based on their retention time and MS spectra with synthetic standards (Fig. 1A-C). Although there was a slight difference in intensity of fragment ions (relative abundance), the major electron ionization ions of 3Z,6Z,9Z-21:H and 3Z,6Z,9Z-23:H were identical to those of synthetic 3Z,6Z,9Z-21:H and 3Z,6Z,9Z-23:H, respectively (Fig. 1B, C). However, 3Z,6Z,9Z-21:H was exclusively found in the male pheromone gland and abdomen, while 3Z,6Z,9Z-23:H was primarily detected in the abdomen of adult females (Fig. 1A).

To accurately quantify the content of these two chemicals, we established a linear regression of each component’s content (x) ranging from 1 ng to 100 ng, plotted against the integrated area (y) in GC/MS (Fig. S1). Consequently, the content of 3Z,6Z,9Z-21:H is significantly higher in the abdomen (125.46 ± 26.28 ng) than in the pheromone gland (16.54 ± 3.35 ng) of adult males (Student’s *t*-test, *P* = 0.008, Fig. 1D). In contrast, 3Z,6Z,9Z-23:H is much more abundant in the abdomen (268.96 ± 36.66 ng) than in the pheromone gland (10.77 ± 3.74 ng) of adult females (Student’s *t*-test, *P* < 0.001, Fig. 1E).

To further investigate the detection of the two candidate type II sex pheromones by male and female moths, we conducted the electroantennogram (EAG) experiments. The results revealed that both 3Z,6Z,9Z-21:H and 3Z,6Z,9Z-23:H elicited strong EAG responses in both male and female moths. As the concentrations of these two components increased from 100 µg to 1 mg, the EAG responses exhibited a significant dose-dependent increase (Fig. 1F). However, electrophysiology responses did not show a significant difference between sexes in response to the same concentration of each chemical, although adults displayed stronger responses to 1 mg of 3Z,6Z,9Z-21:H (Fig. 1F). In contrast, 100 µg of Z11-16:Ald, Z9-16:Ald, and Z9-14:Ald elicited stronger EAG responses in males compared to females, as expected (Fig. 1F).

### Screening and characterization of the receptor for candidate type II sex pheromone in *H. armigera*

**Fig. 2:**
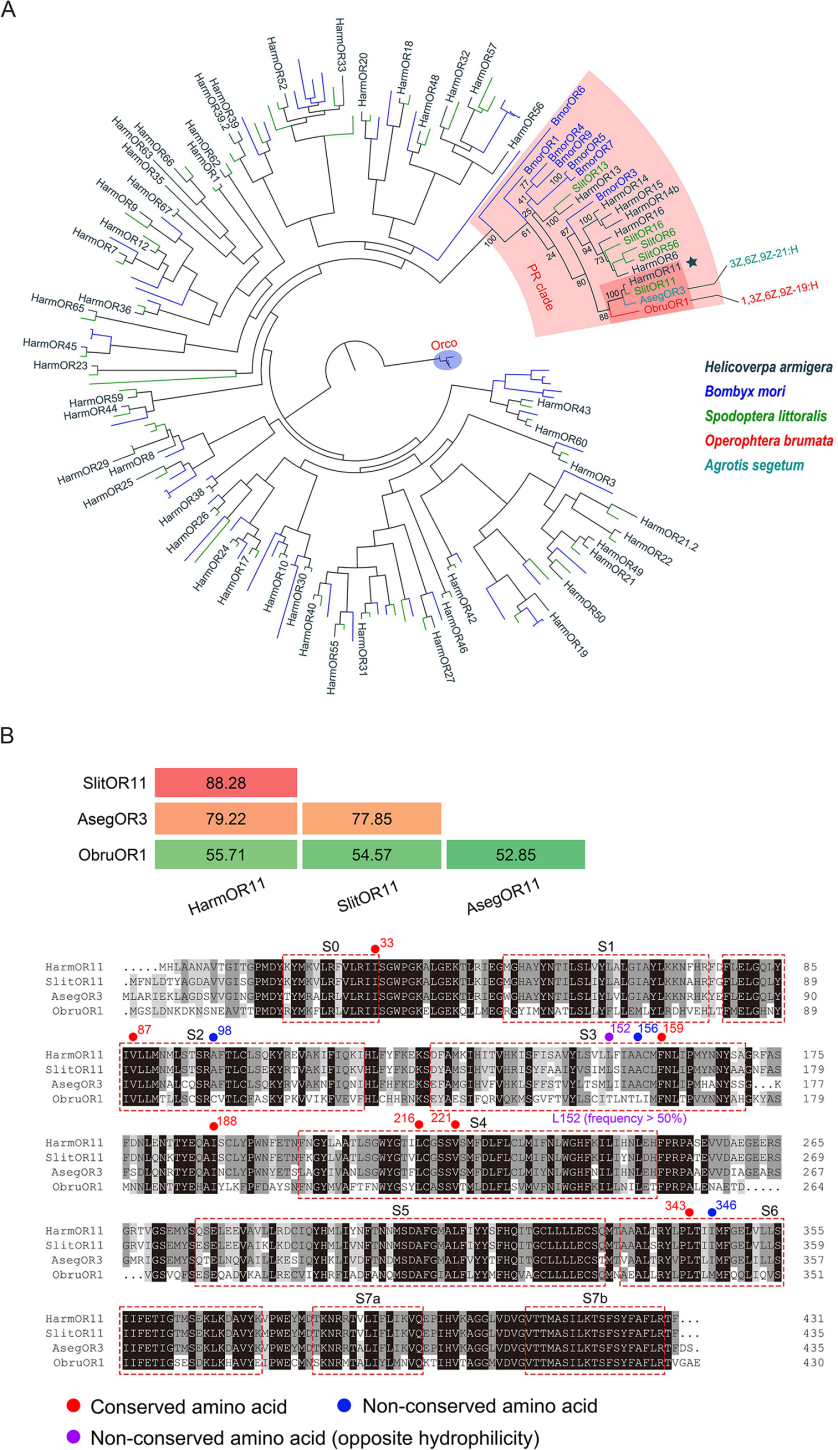
Phylogenetic analysis of odorant receptors in Lepidopteran moths and sequence alignment of HarmOR11 and its orthologous genes. **A** The phylogenetic tree was constructed using RAxML version 8 with the Jones–Taylor–Thornton (JTT) amino acid substitution model [44]. Node support was assessed using a bootstrap method based on 1000 replicates. The phylogenetic tree was constructed using a total of 183 OR sequences from five Lepidoptera species. Dendrograms were created and colour labeled with FigTree v1.4 software. Harm, *Helicoverpa armigera* (dark teal); Bmor, *Bombyx mori* (blue); Slit, *Spodoptera littoralis* (green); Obru, *Operophtera brumata* (red); Aseg, *Agrotis segetum* (teal). The conserved Orco clade was marked in light blue, and the pheromone receptor clade and OR11 subclade were marked in light pink and coral, respectively. **B** Identities and sequence alignment of amino acid sequences of HarmOR11, SlitOR11, AsegOR3, and ObruOR1. The *α*-helix structures of these proteins are highlighted with red dashed boxes. The amino acids involved in ligand binding in HarmOR11 are marked according to the conservation among OR11 lineage

Until now, only one receptor, ObruOR1, in the winter moth, *O. brumata*, and its orthologue, AsegOR3, in *A. segetum*, have been identified as receptors for polyunsaturated polyenes without epoxy or other functional groups [27]. To determine the receptors responsible for the recognition of candidate type II sex pheromones in *H. armigera*, we constructed a phylogenetic tree to identify potential candidates. The phylogenetic tree revealed that ObruOR1 and AsegOR3 clustered within the traditional pheromone receptor clade (highlighted in light pink) (Fig. 2A). Furthermore, *HarmOR11* was identified as the orthologous gene of *ObruOR1* and *AsegOR3* (Fig. 2A), making it the most promising candidate receptor for type II sex pheromones. Sequence alignment results indicated that HarmOR11 shares 55.71% and 79.22% of amino acid identity with ObruOR1 and AsegOR3, respectively (Fig. 2B).

**Fig. 3:**
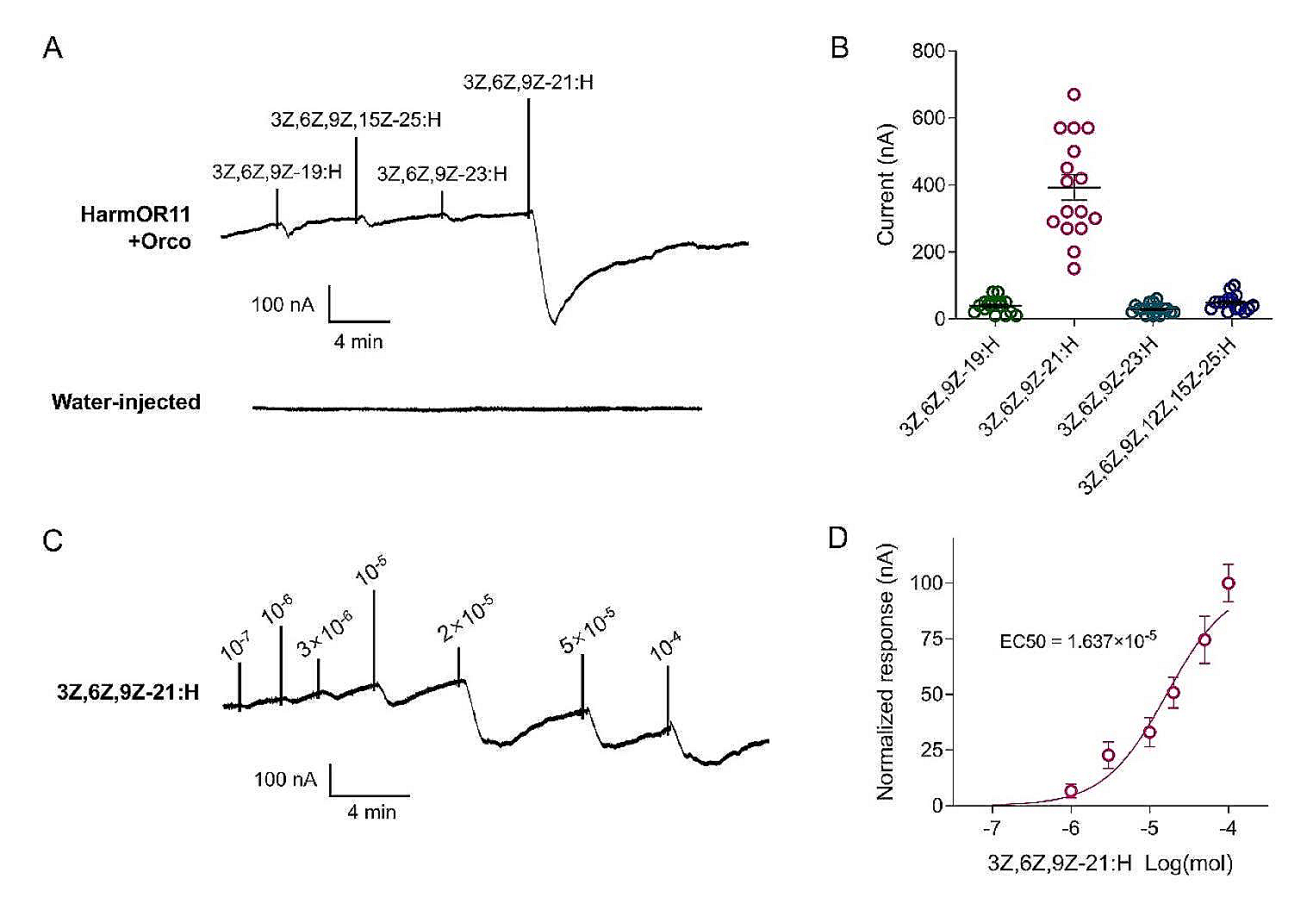
Responses of *Xenopus* oocytes co-expressing HarmOR11/Orco to type II pheromones. **A** Inward current responses of HarmOR11/Orco co-expressing *Xenopus* oocytes exposed to four type II pheromones with a concentration of 10^− 4^ mol. The water-injected oocytes were used as a control. **B** Response profiles of HarmOR11/Orco to four type II pheromones with a concentration of 10^− 4^ mol. Data are shown as mean ± SEM (*n* = 16 biological replicates). **C** HarmOR11/Orco co-expressing *Xenopus* oocytes stimulated with various concentrations of 3Z,6Z,9Z-21:H. **D** Dose response curves of HarmOR11/Orco to 3Z,6Z,9Z-21:H. Error bars indicate the SEM (*n* = 7 biological replicates). The EC_50_ value was 1.637 × 10^− 5^ mol

Subsequently, we conducted functional characterization of HarmOR11 in the *Xenopus* oocyte heterologous expression system, coupled with the TEVC technique. The results revealed that oocytes co-expressing HarmOR11/Orco exhibited robust TEVC responses to 3Z,6Z,9Z-21:H at a concentration of 10^− 4^ mol, while they did not respond or exhibited only very weak responses to the other three polyenes (Fig. 3A, B). Water-injected oocytes showed no responses to any of the tested chemicals (Fig. 3A). Importantly, the responses of HarmOR11/Orco-injected oocytes to 3Z,6Z,9Z-21:H were concentration-dependent, with a threshold lower than 10^− 7^ mol, and an EC_50_ value of 1.637 × 10^− 5^ mol (Fig. 3C, D).

### *HarmOR11* mutants severely reduced the EAG responses to the candidate type II sex pheromones

**Fig. 4:**
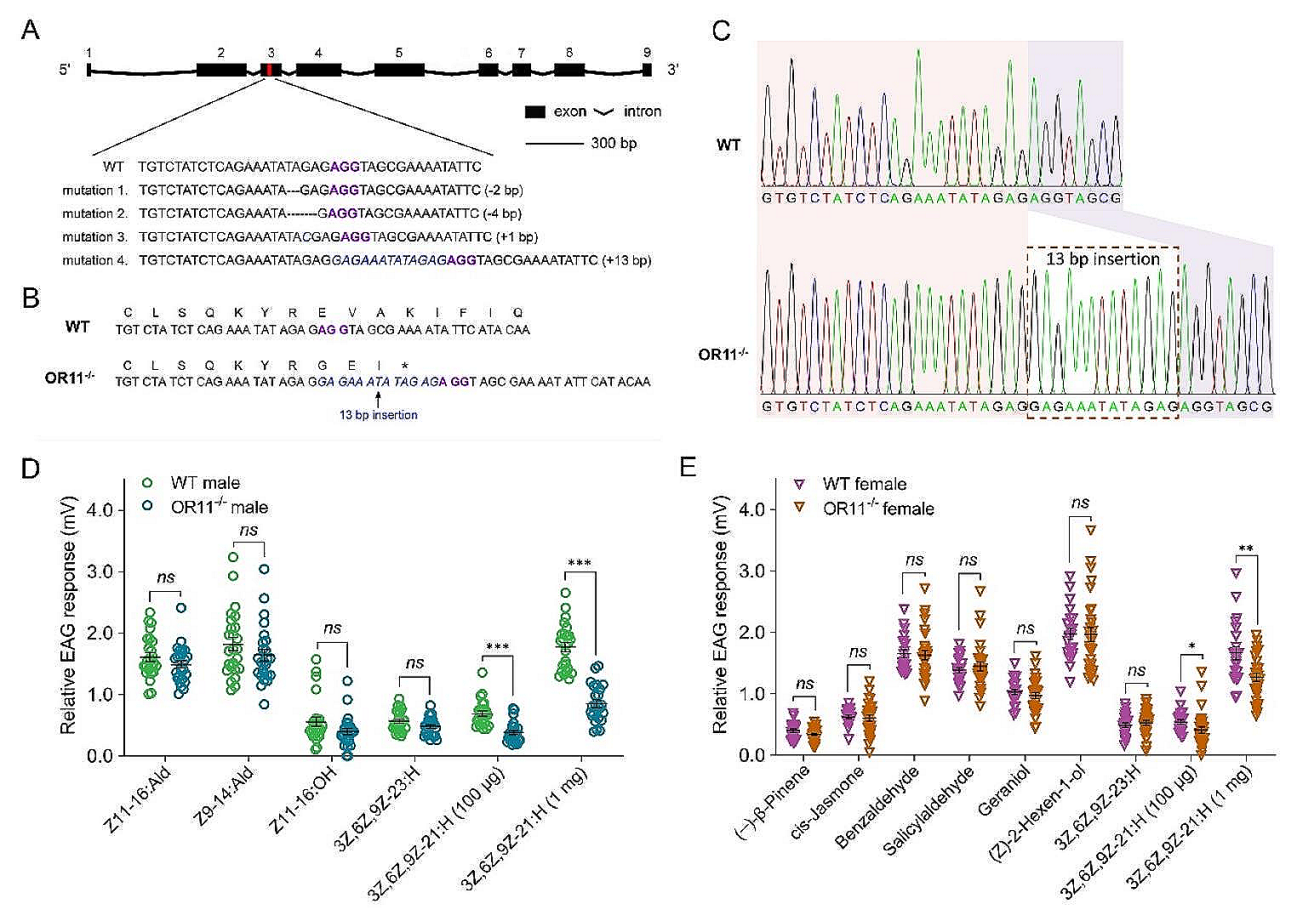
HarmOR11 mediated electrophysiology responses to type II pheromones. **A** HarmOR11 possesses nine exons (black rectangle) in the genome, and a signal guide RNA (sgRNA) target (in red) located in exon 3 was chosen for CRISPR/Cas9-directed gene editing. PAM site (AGG) was marked in purple, and four types of somatic mutations at the targeted loci were obtained. **B** The mutation with a 13-bp DNA fragment insertion caused a frameshift at codon 108 and introduced a premature stop codon, giving rise to a truncated protein of 110 amino acids. **C** The two sequencing chromatograms indicated two genotypes, wild type (WT, top) and *HarmOR11* mutant (bottom), respectively, and the insertion fragment was shown with a rust dushed frame. **D** Relative EAG responses to three type I and two type II pheromones in males of WT (green) and *HarmOR11* mutation (teal). Type I pheromones and 3Z,6Z,9Z-23:H were used in a concentration of 10 µg µL^− 1^, and 3Z,6Z,9Z-21:H was used in concentrations of 10 µg µL^− 1^ and 100 µg µL^− 1^. Data are shown as mean ± SEM (*n* = 25 biological replicates), and statistical analysis was conducted by a two-tailed unpaired Student’s *t*-test. ns, *P* > 0.05; ***, *P* < 0.001. **E** Relative EAG responses to six plant volatiles and two type II pheromones in WT (purple) and *HarmOR11* mutation (rust) females. Plant volatiles and 3Z,6Z,9Z-23:H were applied in a concentration of 10 µg µL^− 1^, and 3Z,6Z,9Z-21:H was used in concentrations of 10 µg µL^− 1^ and 100 µg µL^− 1^. Data are shown as mean ± SEM (*n* = 22 and 30 biological replicates), and were analyzed by a two-tailed unpaired Student’s *t*-test. ns, *P* > 0.05; *, *P* < 0.05; **, *P* < 0.01

To confirm whether *HarmOR11* is the receptor responsible for detecting the candidate type II sex pheromones in vivo, we employed the CRISPR/Cas9 technique to knock out the *HarmOR11* gene. We designed a single-guide RNA (sgRNA) targeting exon 3 with a conserved sequence of 5’-TCTATCTCAGAAATATAGAGAGG**-**3’ to target the gene (Fig. 4A). The nucleotide sequence of the sgRNA was highly specific, particularly the 12 bp sequence (seed region) near the PAM site, which could not be fully matched to other genomic regions, indicating a very low risk of off-target effects. PCR amplification of the fragment carrying the target site from the genomic DNA (gDNA) of each injected G0 moth revealed several types of somatic mutations at the targeted loci of the *HarmOR11* gene (Fig. 4A). Ultimately, we retained the fourth mutation strain due to its largest population. In this strain, a 13-bp short DNA fragment was inserted into the genome before the PAM sequence (Fig. 4A-C). This mutation resulted in a frameshift at codon 108 and introduced a premature stop codon, leading to the production of a truncated protein consisting of 110 amino acids (Fig. 4B).

Given that the both candidate type II sex pheromones elicited strong electrophysiological responses in male and female moths (Fig. 1F), we aimed to confirm whether the *HarmOR11* mutant affected the responses to these pheromones by conducting EAG assays. The results revealed that the EAG responses to 100 µg and 1 mg of 3Z,6Z,9Z-21:H were significantly reduced in male mutants (Fig. 4D). There was no significant difference in the responses to 100 µg of 3Z,6Z,9Z-23:H between wild type and mutated males (Fig. 4D). As expected, the *HarmOR11* mutants did not alter the EAG responses to type I sex pheromones, including Z11-16:Ald, Z9-14:Ald, and Z11-16:OH (Fig. 4D).

A similar situation was observed in females. The EAG responses to 100 µg and 1 mg of 3Z,6Z,9Z-21:H were all significant reduced in female mutants. However, the responses to 100 µg of 3Z,6Z,9Z-23:H and several plant volatiles, including (−)-β-Pinene, cis-Jasmone, Benzaldehyde, Salicylaldehyde, Geraniol, and (Z)-2-Hexen-1-ol, remained unchanged in *HarmOR11* mutated female (Fig. 4E).

### *HarmOR11* mutants impaired the electrophysiology responses to 3Z,6Z,9Z-21:H in sensilla trichoid

**Fig. 5:**
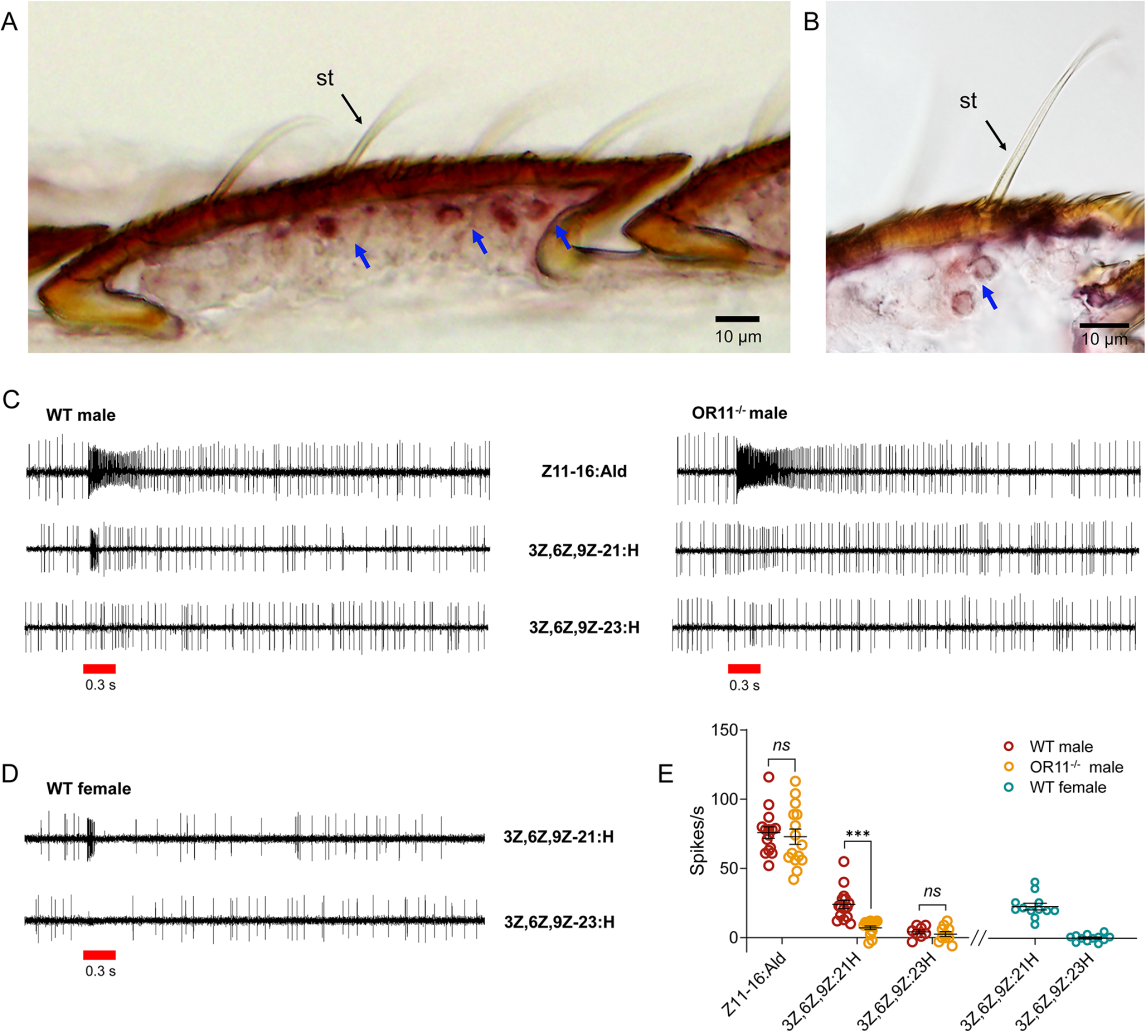
*HarmOR11* mutant impairs the electrophysiology responses to type II pheromones in sensilla trichoid of adults. **A, B** In situ hybridization of *HarmOR11* in female antennae of *Helicoverpa armigera*. In situ hybridization was performed with digoxigenin-labelled antisense RNA probes on longitudinal tissue sections of antennae. Signals were visualized using an anti-DIG antibody. A, the signal in short sensilla trichoid (st); B, the signal in long sensilla trichoid (st). **C** Representative traces of HarmOR11-expressing sensilla trichoid stimulated with 1 mg of pheromone components, including Z11-16:Ald, 3Z,6Z,9Z-21:H, and 3Z,6Z,9Z-23:H in WT and *HarmOR11* mutant males. The red line represents the 0.3 s odor stimulation. **D** Representative traces of HarmOR11-expressing sensilla trichoid activated by 1 mg of 3Z,6Z,9Z-21:H and 3Z,6Z,9Z-23:H in WT and *HarmOR11* mutant females. **E** Quantification of the mean responses to the indicated stimulus for the experiment shown in C and D. Data are plotted as mean ± SEM (*n* = 12–15 biological replicates), and statistical analysis was conducted by a two-tailed unpaired Student’s *t*-test. ns, *P* > 0.05; ***, *P* < 0.001

Previous evidence suggested that *HarmOR11* is expressed in neighboring ORNs with *HarmOR13* within the same sensilla trichoid in males of *H. armigera* [39]. However, the cellular localization of *HarmOR11* in the female antenna has not been determined. To address this, we performed in situ hybridization on cryosections of female *H. armigera* antennae. Longitudinal sections through female antennae were labelled using digoxigenin (DIG)-labelled *HarmOR11* antisense RNA. The results indicated strong *HarmOR11* hybridization signals restricted to the bases of sensilla trichoid in the female antenna (Fig. 5A, B).

To investigated whether the neurons expressing *HarmOR11* respond to the candidate type II sex pheromone, we conducted single-sensillum recordings (SSR) using sensilla trichoid on adult antennae. Previously, *HarmOR11* was reported to be expressed with *HarmOR13* in neighboring ORNs within the same sensilla trichoid in male adults [39]. Therefore, we used Z11-16:Ald to identify the sensilla trichoid housing *HarmOR11* and *HarmOR13* neurons. We tested a total of 132 sensilla trichoid, and 83 of them were strongly activated by Z11-16:Ald, indicating the presence of *HarmOR13* and *HarmOR11*. Remarkably, all these activated sensilla trichoid responded rapidly to 3Z,6Z,9Z-21:H but did not respond to 3Z,6Z,9Z-23:H, even when presented at a total amount of 1 mg (Fig. 5C). However, in *HarmOR11* mutant males, we observed that the sensilla trichoid responding to Z11-16:Ald significantly impaired electrophysiology responses to 3Z,6Z,9Z-21:H (Student’s *t*-test, *P* < 0.001), while they had no effect on the response intensity to Z11-16:Ald (Student’s *t*-test, *P* = 0.667, Fig. 5C, E). Additionally, these sensilla in male mutants showed no response to 3Z,6Z,9Z-23:H, similar to the wild type (Fig. 5C).

In females, *HarmOR11* also specifically expressed in sensilla trichoid (Fig. 5A, B). Therefore, we investigated the responses of the sensilla trichoid to 3Z,6Z,9Z-21:H in the female antenna. It is important to note that there was no chemical marker like Z11-16:Ald in males to locate the sensilla housing *HarmOR11* neurons. Consequently, we swept the sensilla trichoid that responded to 3Z,6Z,9Z-21:H blindly. We tested a total of 153 sensilla trichoid, and 68 of them narrowly tuned to 3Z,6Z,9Z-21:H in a similar manner to that in males (Fig. 5D, E). Not surprisingly, these sensilla exhibited no response to 3Z,6Z,9Z-23:H (Fig. 5D, E). In contrast, we tested 113 sensilla trichoid in antennae of female mutants, but none of them exhibited specific responses to 3Z,6Z,9Z-21:H as observed in the wild type.

### Molecular modeling and simulations uncover the molecular basis of selective recognition of different pheromone types

**Fig. 6:**
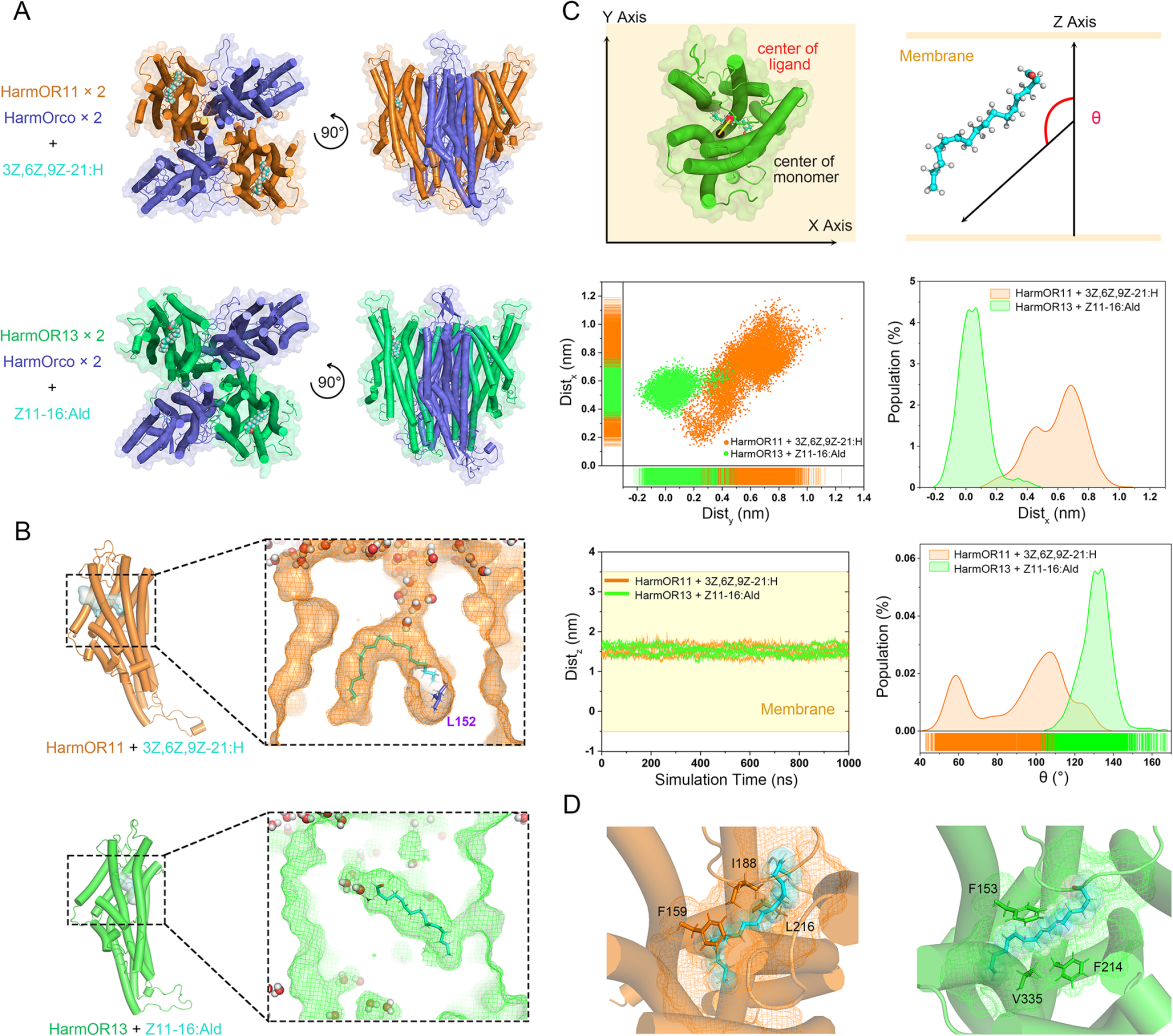
Molecular dynamics simulations reveal distinct binding modalities for pheromones in HarmOR11 and HarmOR13 complexes. **A** Structural models of (HarmOrco)_2_/(HarmOR11)_2_ with 3Z,6Z,9Z-21:H and (HarmOrco)_2_/(HarmOR13)_2_ with Z11-16:Ald. HarmOrco, HarmOR11, HarmOR13 are colored blue, orange, and cyan respectively. **B** Representative ligand binding modes derived from MD simulations, with density maps displaying pheromone conformational distributions and water molecules depicted as spheres. The purple sticks indicate HarmOR11 residue L152, a potential determinant of substrate selectivity. **C** Simulation trajectories and conformational distributions of ligand-receptor center distance components (Dist_x_, Dist_y_, and Dist_z_) and ligand-membrane angle (θ). **D** Key receptor residues interacting with 3Z,6Z,9Z-21:H (HarmOR11, left) and Z11-16:Ald (HarmOR13, right) shown as sticks

To elucidate the divergent recognition mechanisms allowing lineage-specific pheromone receptors to detect distinct sex pheromones, we compared the binding pockets of HarmOR11 and HarmOR13 using computational modeling. Though the exact stoichiometry remains unknown, previous studies put forth a hypothetical 2:2 complex between odorant receptors and Orco proteins [61]. Building on our previous modeling of HarmOR14b-HarmOrco and HarmOR16-HarmOrco heterotetramers [42], we constructed analogous complexes of HarmOR11-HarmOrco and HarmOR13-HarmOrco docking with their corresponding pheromone ligands, 3Z,6Z,9Z-21:H and Z11-16:Ald. We then carried out microsecond-long molecular dynamics simulations of the resulting ligand-receptor complexes to characterize the binding modes and key stabilizing interactions underpinning pheromone recognition (Fig. 6A).

Our MD simulations revealed stable binding of both 3Z,6Z,9Z-21:H and Z11-16:Ald in their respective pockets, albeit with distinct binding modes. Z11-16:Ald points towards the solvent with its aldehyde head group, while 3Z,6Z,9Z-21:H conceals its two heads deeply, likely due to its high hydrophobicity (Fig. 6B, C). Analysis of distance components (Dist_x_, Dist_y_, Dist_z_) between the ligand center and the OR pocket center, as well as the angle (θ) between the ligand and the membrane normal, revealed a considerably broader conformational distribution for 3Z,6Z,9Z-21:H in HarmOR11 compared to Z11-16:Ald in HarmOR13 (Fig. 6C, S2, S3).

Further scrutiny of atomic details unveiled the significant stability of 3Z,6Z,9Z-21:H in HarmOR11, primarily attributed to L216 in transmembrane helix 4 (TMH4), F159 in TMH3, and I188 in extracellular loop 2 (Figs. 2B and 6D, S4). In the parallel investigation of the HarmOR13-Z11-16:Ald system, simulations indicated that the aldehyde group of Z11-16:Ald was solvent-exposed, while the long carbon chain established interactions with surrounding hydrophobic residues (Fig. 6B). Notably, this interaction was predominantly facilitated by F153 in TMH3, F214 in TMH4, and V335 in TMH6 (Fig. 6D, S4).

Additionally, our sequence alignment analysis highlighted L152, a specific residue in HarmOR11 located at one end of the crescent-shaped pocket (Fig, 2B, 6B), as a potential key factor influencing ligand selectivity. Notably, at the corresponding position in AsegOR3, which recognizes the same ligand (3Z,6Z,9Z-21:H), a similarly hydrophobic leucine is present (Fig. 2B). In contrast, in the pocket of ObruOR1, which recognizes the relatively less hydrophobic 1,3Z,6Z,9Z-19:H, this position is occupied by a polar threonine. These sequence distinctions likely play a role in determining ligand selectivity.

**Fig. 7:**
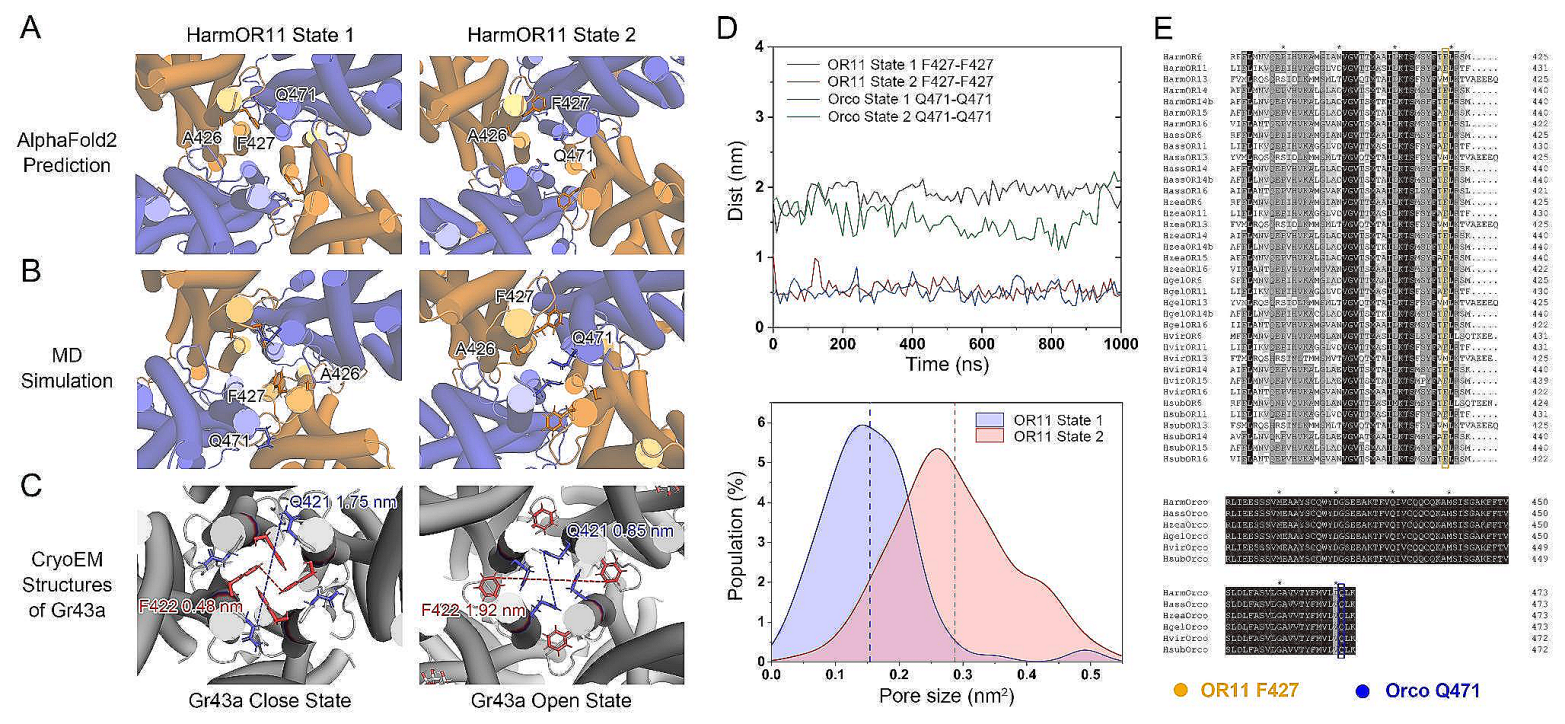
Key residues involved in channel opening as predicted by AlphaFold2 and observed in MD simulations. **A** AlphaFold2 predictions showing two conformational states of the ion channel in the HarmOR11-Orco heterotetramer. State1 is a closed state with F427 of HarmOR11 oriented towards the channel center, and State2 is a putative open state with F427 of HarmOR11 moving away from the center while Q471 of HarmOrco moving towards the center. **B** Representative conformations from MD simulations initiated from both State1 and State2. **C** Conformational transitions of key residues F422 and Q421 in the closed and open states of the gustatory receptor GR43a, as revealed by recently published cryo-EM structures (PDB ID: 8JM9 and 8X83). **D** Trajectories of the distances between the F427 residues of HarmOR11 and the Q471 residues, alongside the population of the pore size (calculated as the area between two F427 and two Q471 residues) from MD simulations of the HarmOR11-Orco heterotetramer in both the apo State 1 and the pheromone-bound State 2. **E** Sequence alignment of pheromone receptors highlighting key amino acids potentially related to ion channel opening

Intriguingly, the AlphaFold2 predictions for the HarmOR11-Orco heterotetramer revealed two distinct conformational states of the critical gating residues F427 of OR11 and Q471 of Orco: a closed state with both residues directed towards the ion channel’s center (State1) and another presumed open state with both residues facing outward (State2, used for simulating the pheromone-bound state), as depicted in Fig. 7A. For the HarmOR13-Orco heterotetramer, all predictions pointed to a uniform closed state, leading us to forego further analysis for this heterotetramer. To further explore the molecular mechanism of channel activation by substrate binding, we conducted additional MD simulations starting from the closed State1 (Fig. 7B) and compared these with simulations of the pheromone-bound states originating from the presumed open State2 (Supplementary Table S3). These simulations showed stability, with State 2 maintaining a larger pore size with large separation of the F427 residues, and a closer arrangement of the Q471 residues (Fig. 7D).

This observation is reminiscent of the substrate binding induced conformational shifts observed in the recently resolved cryo-EM homotetrameric structures of gustatory receptors (Gr), such as *Drosophila melanogaster* Gr43a (DmGr43a) and *B. mori* Gr9 (BmGr9) in both close and open states [62–64], which facilitate ion passage by rotating helices and repositioning the side chains of hydrophobic (F422 in Gr43a, F444 in BmGr9) and hydrophilic residues (Q421 in Gr43a, Q443 in BmGr9) at the entrance of the channel (Fig. 7C). Notably, the position preceding F427 in HarmOR11 is occupied by a smaller residue, Ala, rather than the hydrophilic Gln. Despite this, we identified similar hydrophilic residues like Q471 in Orco. We hypothesize that the smaller size of Ala may facilitate channel opening. Thus, we propose that the ion channel opening mechanism in HarmOR11 might slightly differ from that in Gr43a, potentially involving rotation of the S7b helix and replacement of F427 with the shorter amino acid A426. Our sequence alignment of typical odorant receptors from six insects suggests that this pattern of replacing F427 with shorter side chains or substituting with hydrophilic amino acids could be somewhat universal (Fig. 7E). However, we did not observe complete ion passage events or spontaneous ion channel openings triggered by pheromone binding, likely because such processes exceed the microsecond time scale of our simulations.

## Discussion

Moth sex pheromones have long been a central focus in the field of insect chemical ecology due to their profound influence on mating behavior and the remarkably specific recognition mechanisms they involve. Sex pheromones are typically categorized into four structural types, and each moth species usually utilizes only one type [6]. However, recent investigations indicated that more moth species than previously thought employ a combination of two pheromone types in their sexual communications [18, 65, 66], notably a blend of type I and type II pheromones. This raises intriguing questions about the transition from the ancestral detection of type I pheromones to type II pheromones and the structural basis of PRs to specifically adapt to the distinct structures of each pheromone type.

In this study, we identified two candidate type II sex pheromones, 3Z,6Z,9Z-21:H and 3Z,6Z,9Z-23:H, in *H. armigera*. These compounds also function as type II sex pheromones in various moth species; for instance, 3Z,6Z,9Z-21:H in *Heliothis virescens* and *Achaea janata* [67, 68], and 3Z,6Z,9Z-23:H in *Rehimena surusalis* and *Tmetolophota atristriga* [18, 19]. Despite exhibiting sexual dimorphism in quantities across tissues, these pheromones elicited comparable EAG responses in both sexes. This finding departs from conventional expectations, challenging the widely accepted notion that the opposite sex of the pheromone emitter typically shows stronger electrophysiological responses to sex pheromones. This observation aligns with the sex-biased expression patterns observed in PR genes [23, 69]. PRs, belonging to a specialized subfamily of ORs, demonstrate a high level of conservation, evident in the distinct clustering of moth PRs within the “PR clade” in phylogenetic trees of moth ORs [27, 70].

Fortunately, a specific PR gene, ObruOR1, and its orthologue have been confirmed to recognize similar pheromones to those identified in *H. armigera* [27]. Phylogenetic analysis and electrophysiological assays verified HarmOR11, a typical PR, as a receptor for 3Z,6Z,9Z-21:H. Notably, HarmOR11, along with its orthologues, has received special attention for its silent responsiveness to type I pheromones in various genera of Noctuidae, including *Helicoverpa* [25, 37, 42], *Heliothis* [71, 72], *Spodoptera* [73–75], *Mythimna* [76, 77], and *Athetis* [78]. Thus, it is reasonable to infer that the OR11 clade is specifically tuned to type II pheromones in Noctuidae moths.

In a recent study, Liu et al. identified two type II sex pheromones, 3Z,6Z,9Z-21:H and 3Z,6Z,9Z,12Z,15Z-23:H, from the hairpencils (referred to as male sex glands in this study) of a Heliothinae moth, *H.* (*Chloridea*) *virescens*. These pheromones elicited strong electrophysiological responses in female antennae [67]. Although their effects on the mating behaviors of adults have not been analyzed, it seems likely that such chemical compounds are widely distributed among Noctuidae moths [18], given the high conservation of *OR11* genes.

Previously, *OR11* has been demonstrated to locate within the same sensilla trichoid with *OR13* in male antennae of *H. armigera*, *H. assulta*, and *H. virescens* [39, 79]. Through SSR assays, we found that the sensilla trichoid housing *HarmOR11* neurons specifically responded to 3Z,6Z,9Z-21:H but not to 3Z,6Z,9Z-23:H in both male and female moths. Traditionally, sensilla trichoid on male antennae have been broadly acknowledged for detecting sex pheromones emitted by conspecific females [25]. Conversely, in females, these sensilla trichoid have been reported to sense plant volatiles [80, 81] or to autodetect pheromones [82]. However, it remains unexpected that nearly half of the sensilla trichoid on both male [39] and female antennae in *H. armigera* respond to 3Z,6Z,9Z-21:H, suggesting the important role of the *HarmOR11* gene. In EAG experiments, the electrophysiological responses to 3Z,6Z,9Z-21:H were significantly weakened but not completely abolished in both male and female mutant strains. This suggests that there may be other ORs that can be activated by 3Z,6Z,9Z-21:H. As expected, the neurons expressing *HarmOR11* in mutants impaired the electrophysiological responses to 3Z,6Z,9Z-21:H in both sexes, implying that HarmOR11 functions to recognize type II pheromones in vivo.

Moth PRs are highly conserved and form a dedicated subfamily of ORs in the phylogenetic trees of moth ORs [69]. Recent studies have revealed novel lineages within OR clades responsible for detecting moth sex pheromones [26, 83–87], indicating multipe orgins of moths PRs. This perspective is resonable given the diverse structures of sex pheromones in moths. However, previous results did not establish solid connections between PR clades and pheromone types. To date, no PRs have reported to recognize type III pheromones, leaving their evolutionary relationships among moth ORs largely unknown. PRs for type 0 pheromones have been identified and characterized in a non-ditrysian moth, *Eriocrania semipurpurella* [83]. Due to the similar chemical structures of its pheromones with plant volatiles, it is understandable that the PRs for type 0 pheromones are not clustered into the novel (indicated by SlitOR5 [84]) or traditional PR clades [83]. Concerning receptors for type I and type II pheromones, they do not exhibit clear seperation into distinct clades. On the contrary, receptors for type I pheromones are found in both the novel [84, 85] and traditional PR clades [41]. The close evolutionary relationships beween PRs for type I and type II pheromones in the traditional PR clade provide an excellent model for studying their structure and function relations.

To unravel the structural mechanisms governing sensory specialization in pheromone receptors, we performed an in-depth comparison of HarmOR11 and the type I PR HarmOR13 using molecular modeling and simulations. Our analysis yields valuable insights into the divergent recognition strategies employed by these lineage-specific receptors. Notably, we identified L152 in HarmOR11 as a potential selectivity filter influencing its unique ligand preferences. By further incorporating our prior findings on type I PRs HarmOR14b and HarmOR16 (Fig. S5) [42], recognizable patterns emerge - type I pheromones appear to utilize hydrophilic head groups to form hydrogen bonds with their receptors, whereas HarmOR11’s type II pheromones are more hydrophobically stabilized in an elongated pocket, suggesting distinct entropy-driven recognition. Synthesizing these observations, we propose a model wherein type I pheromones employ polar moieties for selective receptor interactions, while type II receptors like HarmOR11 adopt more hydrophobic binding sites fitting their non-polar pheromones. These specialized recognition strategies deepen our understanding of the diverse modes of selectivity governing these intricate chemosensory systems.

The AlphaFold2 predictions identified two conformational states for the HarmOR11-Orco heterotetramer: a closed state with potential gating residue F427 of OR11 facing the ion channel’s center and an open state with F427 of OR11 moved away from the center and Q471 of Orco rotated towards the center. Additional MD simulations starting from both states showed that the open state maintained a larger pore size, significant separation of the F427 residues, and closer proximity of the Q471 residues, reminiscent of substrate-induced conformational shifts seen in gustatory receptors like *D. melanogaster* Gr43a and *B. mori* Gr9. These conformational shifts might facilitate ion passage by altering the position of key hydrophobic and hydrophilic residues. Interestingly, the position before F427 in HarmOR11, occupied by a smaller residue, Ala. Furthermore, a comprehensive sequence alignment across typical pheromone receptors suggests a potentially universal mechanism for channel opening, involving the rotation of the pore helix S7b to replace hydrophobic Phe residues with shorter or hydrophilic ones facing the channel, thereby facilitating channel opening. Despite these valuable findings, the simulations did not capture spontaneous ion channel openings, highlighting the need for more prolonged simulations or experimental models to fully understand ion channel dynamics. We are dedicated to tackling these challenges in our future research.

In summary, our findings not only reveal the presence and recognition mechanism of type II pheromones in a noctuid moth, but also provide a structural framework of PRs, enhancing our comprehension of the connections between evolutionary adaptations and the emergence of new pheromones types.

### Electronic supplementary material

Below is the link to the electronic supplementary material.

Supplementary Material 1

## Data Availability

All data generated or analyzed during this study are included in this article (and its Supplementary files).

## Abbreviations

OR: Odorant receptor
ORN: Olfactory receptor neuron
PR: Pheromone receptor
Orco: Odorant receptor co-receptor
GC/MS: Gas chromatography and mass spectrometry
EI: Electron ionization
PG: Pheromone gland
EAG: Electroantennogram
TEVC: Two-electrode voltage-clamp
cRNA: caped RNA
SSR: Single-sensillum recordings
JTT: Jones–Taylor–Thornton
sgRNA: single-guide RNA
TMH: Transmembrane helix
MD: Molecular dynamics
DSD: Degenerate Sequence Decoding
CGenFF: CHARMM general force field
