# Supplementary material for: The novel function of an orphan pheromone receptor reveals the sensory specializations of two potential distinct types of sex pheromones in noctuid moth
Source: Cell Mol Life Sci. 2024 Jun 15;81(1):259. doi: 10.1007/s00018-024-05303-2 (PMC11335300; doi:10.1007/s00018-024-05303-2)

Supporting Information

The novel function of an orphan pheromone receptor reveals the sensory specializations of two potential distinct types of sex pheromones in noctuid moth

Chenrui Wang[1,#], Song Cao[2,3,#], Chen Shi[4,#], Mengbo Guo[1,5], Dongdong Sun[1], Zheyi Liu[6], Peng Xiu[4], Yong Wang[6,7,*], Guirong Wang[1,2,*], Yang Liu[1,*]

[1]State Key Laboratory for Biology of Plant Diseases and Insect Pests, Institute of Plant Protection, Chinese Academy of Agricultural Sciences, Beijing 100193, China
[2]Shenzhen Branch, Guangdong Laboratory for Lingnan Modern Agriculture, Genome Analysis Laboratory of the Ministry of Agriculture and Rural Affairs, Agricultural Genomics Institute at Shenzhen, Chinese Academy of Agricultural Sciences, Shenzhen 518120, China
[3]Institute of Evolution and Ecology, School of Life Sciences, Central China Normal University, Wuhan 430079, China
[4]Department of Engineering Mechanics, Zhejiang University, Hangzhou 310027, China
[5]Department of Plant Protection, Advanced College of Agricultural Sciences, Zhejiang A & F University, Hangzhou 311300, China
[6]College of Life Sciences, Zhejiang University, Hangzhou 310058, China
[7]The Provincial International Science and Technology Cooperation Base on Engineering Biology, International Campus of Zhejiang University, Haining 314499, China

[#]These authors contributed equally

[*]Correspondence:
Yang Liu
yangliu@ippcaas.cn

Guirong Wang
wangguirong@caas.cn

Yong Wang
yongwang_isb@zju.edu.cn

Table S1 Type II sex pheromones used in this study.

**Chemical component**	**Short name**	**Purity**	**CAS number**
3Z,6Z,9Z-nonadecatriene	3Z,6Z,9Z-19:H	95.3%	89353-62-8
3Z,6Z,9Z-heneicosatriene	3Z,6Z,9Z-21:H	93.6%	87255-15-0
3Z,6Z,9Z-tricosatriene	3Z,6Z,9Z-23:H	94.5%	102673-51-8
3Z,6Z,9Z,12Z,15Z-pentacosapentaene	3Z,6Z,9Z,12Z,15Z-25:H	73.6%	854201-96-0

Table S2 Primers used in this study.

**Primer**	**Forward (5' to 3')**	**Reverse (5' to 3')**
**Primers for gene clone**		
HarmOrco	ATGATGACCAAGGTGAAGGC	TTACTTGAGCTGTATCAATACC
HarmOR11	ATGCATCTTGCAGGCAATGC	TTAAAACGTGCGTAGAAAAGC
**Primers for pT7Ts vector construction**		
HarmOrco	TCAGATATCGCCACCATGATGACCAAGGTGAAGGC	TCAGCATGCTTACTTGAGCTGTATCAATACC
HarmOR11	TCAGGGCCCGCCACCATGAGCTTTAAAAAATTTC	TCAGCGGCCGCTTAAAACGTGCGTAGAAAAGC
**Primers for *in situ* hybridization**		
HarmOR11	TCAAAGCTTTTCTGGAGCTGGGACAGTTG	TCAGAATTCTTTCACTCATAGTGCCGATGG
**Primers for OR11 sgRNA synthesis and mutant screening**		
OR11-sgRNA	GAAATTAATACGACTCACTATAGCTATCTCAGAAATATAGAG	TTCTAGCTCTAAAACCTCTATATTTCTGAGATAGC
OR11-mutant	CTGGAGCTGGGACAGTTGTATATTG	TGAGACAGAGGAATAAGTCAAACATCG

Table S3 Information for molecular dynamic stimulations in this study.

Molecular dynamic simulations		
**System**	**Ligand**	**MD times**
HarmOR11 (State 1) × 2 : HarmOrco × 2	apo	1000 ns × 2
HarmOR11 (State 2) × 2 : HarmOrco × 2	3Z,6Z,9Z-21:H	1000 ns × 2
HarmOR13 × 2: HarmOrco ×2	apo	1000 ns × 2
HarmOR13 × 2: HarmOrco × 2	Z11-16:Ald	1000 ns × 2

Fig. S1 Linear regression analysis between the contents and the integrated areas of Z3,Z6,Z9–21:H and Z3,Z6,Z9–23:H in GC/MS.

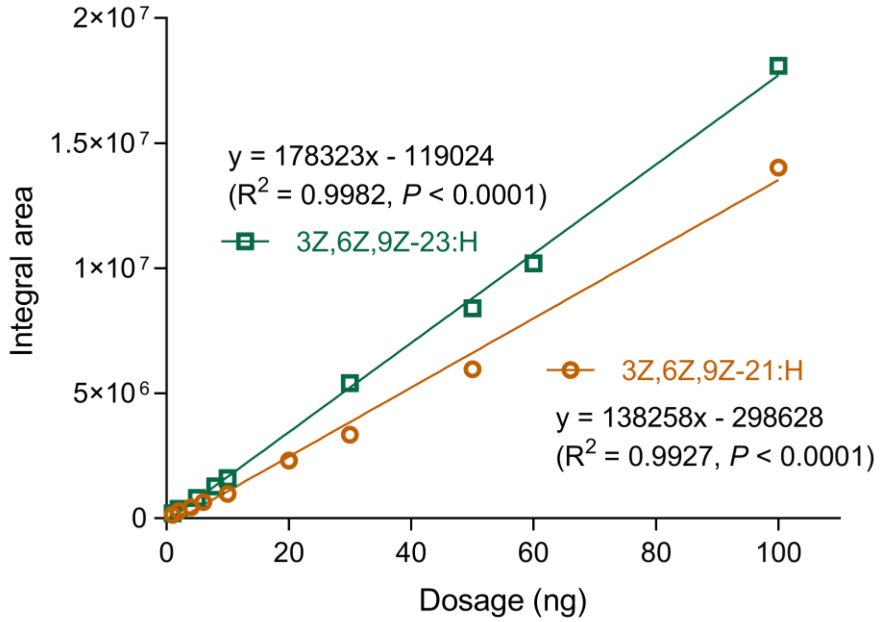

Fig. S2 Molecular Dynamics Simulations of $(HarmOrco)_2/(HarmOR11)_2$ with 3Z,6Z,9Z-21:H. Each simulation involved two replicas of the pheromone-bound OR, resulting in a total of four replicas from two one-microsecond MD simulations. The figure shows trajectories and conformational distributions of ligand-receptor center distance components ($Dist_x$, $Dist_y$, and $Dist_z$) and ligand-membrane angle (θ). Additionally, two representative binding modes are shown.

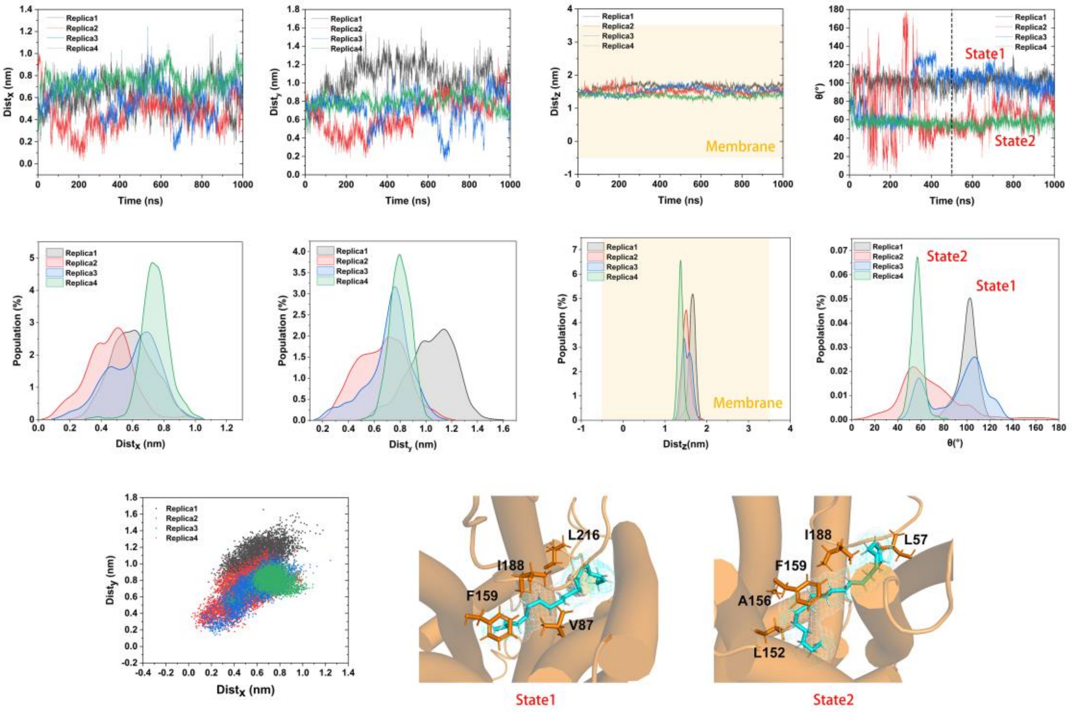

Fig. S3 Molecular Dynamics Simulations of $(HarmOrco)_2/(HarmOR13)_2$ with Z11-16:Ald. Each simulation involved two replicas of the pheromone-bound OR, resulting in a total of four replicas from two one-microsecond MD simulations. The figure shows trajectories and conformational distributions of ligand-receptor center distance components ($Dist_x$, $Dist_y$, and $Dist_z$) and ligand-membrane angle (θ).

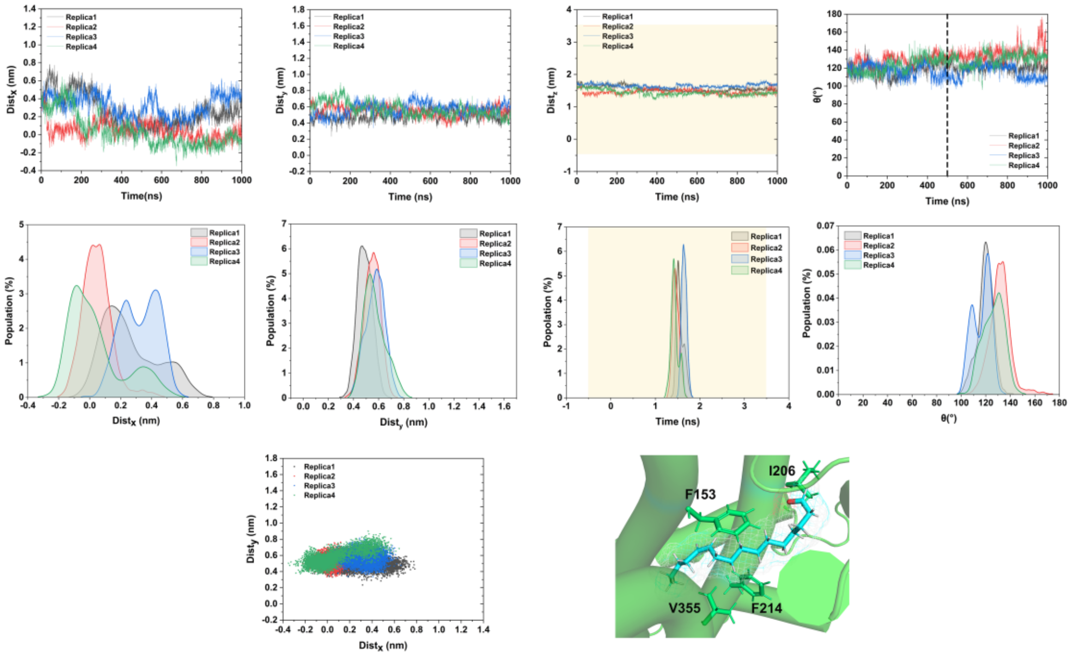

Fig. S4 Comparison of the transmembrane structures and interaction frequencies of HarmOR11 and HarmOR13 model. A and B) The key amino acid residues involved in ligand binding are marked in red and green in HarmOR11 and HarmOR13, respectively. C and D) Decimals surrounding the circle show the ratios of the time that each interaction exists to the whole MD simulation time. Only the ratios above 10% have been listed.

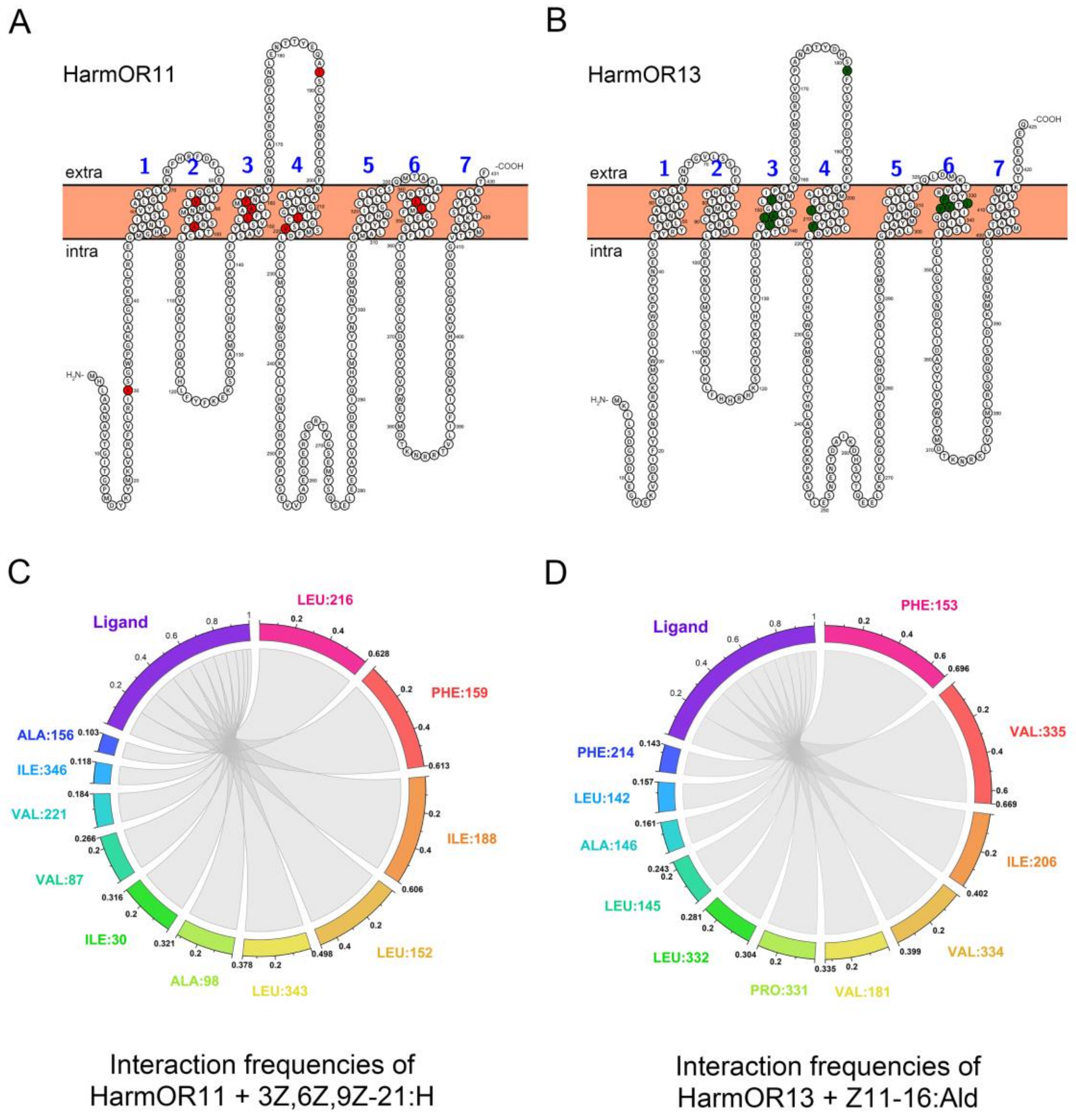

Fig. S5 Representative binding modes and conformational distributions of ligand-receptor center distance components ($Dist_x$, $Dist_y$), ligand-membrane angle (θ), and radius of gyration of ligand (R_g) in the pheromone-bound OR systems. The simulation data of HarmOR14b and HarmOR16 were obtained from our previous work.

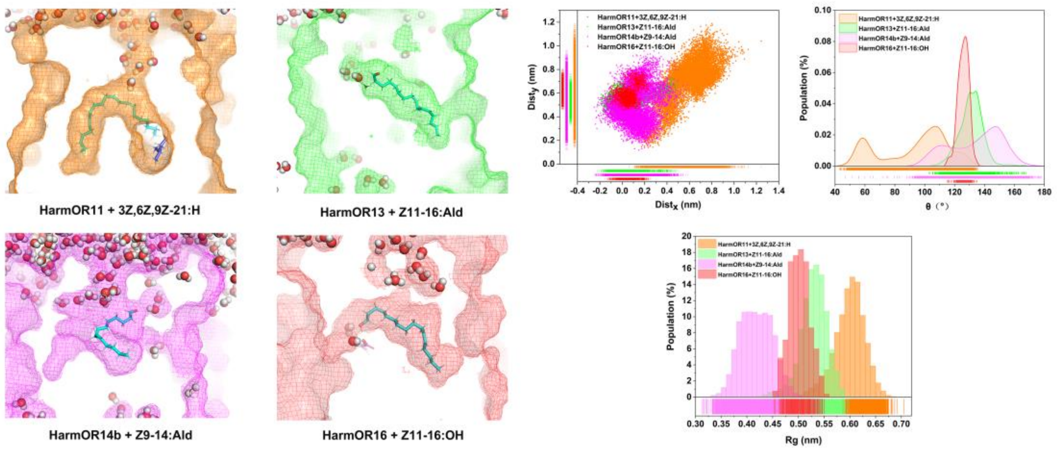

Supplement: Supplementary file 1 — Supplementary Material 1 [file 18_2024_5303_MOESM1_ESM.pdf]
